# Mapping the Pareto Optimal Design Space for a Functionally Deimmunized Biotherapeutic Candidate

**DOI:** 10.1371/journal.pcbi.1003988

**Published:** 2015-01-08

**Authors:** Regina S. Salvat, Andrew S. Parker, Yoonjoo Choi, Chris Bailey-Kellogg, Karl E. Griswold

**Affiliations:** 1 Thayer School of Engineering, Dartmouth, Hanover, New Hampshire, United States of America; 2 Department of Computer Science, Dartmouth, Hanover, New Hampshire, United States of America; 3 Program in Molecular and Cellular Biology, Dartmouth, Hanover, New Hampshire, United States of America; Imperial College London, United Kingdom

## Abstract

The immunogenicity of biotherapeutics can bottleneck development pipelines and poses a barrier to widespread clinical application. As a result, there is a growing need for improved deimmunization technologies. We have recently described algorithms that simultaneously optimize proteins for both reduced T cell epitope content and high-level function. *In silico* analysis of this dual objective design space reveals that there is no single global optimum with respect to protein deimmunization. Instead, mutagenic epitope deletion yields a spectrum of designs that exhibit tradeoffs between immunogenic potential and molecular function. The leading edge of this design space is the Pareto frontier, i.e. the undominated variants for which no other single design exhibits better performance in both criteria. Here, the Pareto frontier of a therapeutic enzyme has been designed, constructed, and evaluated experimentally. Various measures of protein performance were found to map a functional sequence space that correlated well with computational predictions. These results represent the first systematic and rigorous assessment of the functional penalty that must be paid for pursuing progressively more deimmunized biotherapeutic candidates. Given this capacity to rapidly assess and design for tradeoffs between protein immunogenicity and functionality, these algorithms may prove useful in augmenting, accelerating, and de-risking experimental deimmunization efforts.

## Author Summary

Protein therapeutics have created a revolution in disease therapy, providing improved outcomes for prevalent illnesses and conditions while at the same time yielding treatments for diseases that were previously intractable. However, this powerful class of drugs is subject to their own unique challenges and risk factors. In particular, the biological origins of therapeutic proteins predispose them towards eliciting a detrimental immune response from the patient's own body. Therefore, fully capitalizing on the medicinal reservoir of natural and engineered proteins will require efficient, effective, and broadly applicable deimmunization technologies. We have developed deimmunization algorithms that simultaneously optimize therapeutic candidates for both low immunogenicity and high-level activity and stability. Here, we combine computational modeling and experimental analysis to show that the process of protein deimmunization manifests inherent tradeoffs between immunogenic potential and biomolecular function. Our experimental results demonstrate that dual objective optimization allows us to assess and design for these tradeoffs, thereby enabling facile construction of deimmunized variants that span a broad range of immunogenicity and functionality performance parameters. Thus, we can rapidly map the design space for deimmunized drug candidates, and we can use this information to guide selection of engineered proteins that are most likely to meet performance benchmarks for a given clinical application.

## Introduction

Therapeutic proteins are revolutionizing disease therapy across a broad range of indications and illnesses, and biotherapeutic sales are an increasingly important part of the pharmaceuticals market [1], [2]. However, these powerful drugs suffer from their own limitations, which, if addressed, could accelerate the pace of biotherapeutic development and approval. A relatively unique risk factor for protein therapeutics is their inherent potential to induce anti-drug immune responses in human patients [3], [4], [5]. These undesirable immune reactions can compromise drug efficacy or cause more serious adverse events [6], [7].

In a healthy human immune system, all extracellular proteins are sampled by antigen presenting cells (APCs). Once internalized by APCs, a protein is cleaved into small peptide fragments, putative immunogenic segments are loaded into the groove of class II major histocompatibility complex proteins (MHC II), and the complexes are trafficked to the APC surface. True immunogenic peptides, termed T cell epitopes, facilitate the formation of ternary MHC II-peptide-T cell receptor complexes with surface receptors of cognate CD4+ T cells [8]. This critical molecular recognition event initiates a signaling cascade that drives stimulation of helper T cells, maturation of B cells, and ultimately production of circulating antibodies that bind to and clear the foreign therapeutic protein. Detailed knowledge of this process enables protein deimmunization via mutation of key residues in immunogenic epitopes, a methodology commonly known as T cell epitope deletion.

There exist in the literature numerous examples of successful T cell epitope deletion projects. To date, the majority of these efforts have relied on time, labor, and resource intensive experimental strategies. Experimentally driven approaches entail dividing the target protein's primary sequence into a large panel of overlapping peptides, synthesizing those peptides, and using them for exhaustive epitope mapping with human peripheral blood mononuclear cells and/or purified human MHC II proteins [9], [10], [11], [12], [13], [14]. To circumvent the considerable effort and expense required for experimental epitope mapping, a wide range of epitope prediction tools may be accessed [15], [16], [17], [18], [19], [20], [21], [22], [23]. In some recent cases, such tools have been leveraged to good effect in deimmunizing therapeutic candidates [24], [25].

Identification and mapping of T cell epitopes is a relatively mature field, but selection of mutations that simultaneously delete epitopes and maintain protein function remains a challenging task. Experimentally driven deimmunization typically relies on alanine scanning or similar empirical strategies to select epitope deleting mutations. While Cantor *et al.* employed epitope prediction to rapidly identify immunogenic regions of asparaginase, their selection of deimmunizing yet function-preserving mutations required construction of large combinatorial protein libraries and implementation of a sophisticated ultra-high throughput screen [24]. As an alternative to the above methods, bioinformatics tools are increasingly used to filter prospective deimmunizing mutations for those least likely to disrupt protein structure and function [12], [26], however these *in silico* analyses of a mutation's structural and functional consequences have historically been applied *post hoc*. Any such sequential application of computational tools fails to consider the combined effects of all mutations on immunogenicity and function, thereby precluding a global approach to protein deimmunization. Thus, while T cell epitope deletion is a well validated methodology, the success, efficiency, and general utility of the approach would be enhanced by bringing to bear more advanced protein engineering and design technologies.

The next generation of protein deimmunization tools have seamlessly integrated immunoinformatic epitope prediction with *in silico* analysis of the functional consequences associated with prospective deimmunizing mutations [27], [28], [29], [30]. By packaging both design objectives in a single optimization algorithm, these technologies enable global protein design and deimmunization on a highly compressed time scale. The first two iterations of these novel algorithms, Dynamic Programming for Deimmunizing Proteins (DP^2^) and Integer Programming for Immunogenic Proteins (IP^2^), have undergone preliminary experimental validation with *Enterobacter cloacae* P99 Beta-lactamase (P99βL) [25], [31], a biotherapeutic that has been deimmunized previously using conventional experimentally-driven techniques [9]. Here we deimmunize the P99βL target using a more advanced extension of IP^2^, embodied in the protein design algorithm “Protein Engineering Pareto Frontier” (Pepfr) [32]. Whereas IP^2^ samples a subset of the designs that optimally balance the immunogenicity and functionality objectives, Pepfr generates the entire set of Pareto optimal variants, i.e. all enzymes whose predicted immunogenicity and functionality are not simultaneously dominated by any other single design. Eighteen of these Pareto optimal variants have been produced and subjected to a rigorous experimental analysis of key performance parameters. This combined computational and experimental analysis of increasingly aggressive plans has provided new insights into the inherent tradeoffs linking the target enzyme's sequence, function, and immunogenic potential. As a whole, this work outlines a design-based approach to functional deimmunization of biotherapeutic candidates.

## Results

### Computational Design

**Figure 1:**
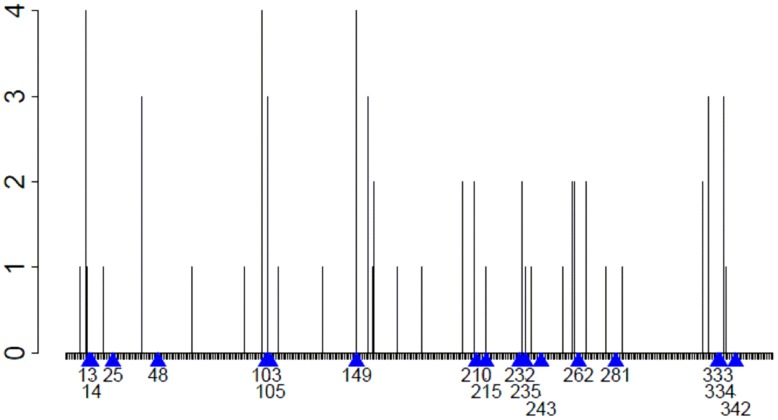
Epitope map of P99βL. The P99βL sequence was analyzed using ProPred set to a 5% threshold. Every nonamer peptide was classified as a binder or non-binder of alleles DRB1*0101, 0401, 0701, and 1501. The number of alleles that bind each nonamer peptide (y-axis) is indicated by a bar at the starting position of the peptide (x-axis). The positions of engineered mutations from the deimmunized enzymes are indicated with blue arrows and residue numbers.

Whereas previous computationally-driven deimmunization of P99βL had targeted eight common MHC II alleles [25], [31], here we optimized against only four alleles (DRB1*0101, 0401, 0701, and 1501) for which MHC II-peptide binding experiments had been fully optimized [33]. Analysis with the ProPred epitope prediction tool [20] revealed that putative immunogenic peptides were broadly distributed throughout the sequence, with several discrete regions exhibiting numerous overlapping and promiscuous epitopes, e.g. proximal to residues 14, 105, 210, 235, and 334 (Fig. 1). While our prior P99βL deimmunization efforts had focused on validation of our protein optimization algorithms, the objective of the current study was a systematic analysis of the sequence-function-immunoreactivity tradeoffs that are inherent to the deimmunization process.

In pursuit of this goal, we applied the Pepfr protein design algorithm [32] to optimize the two objective functions derived from the IP^2^ deimmunization formulation [28]: a sequence score (*S^seq^*, materials and methods equation 1), capturing the predicted effects of mutations on protein function, and an epitope score (*S^epi^*, materials and methods equation 2), capturing the predicted effects of mutations on protein immunogenicity. Both scores are defined such that lower is better (less perturbation to function and reduced immunogenicity, respectively). Pepfr identifies the “Pareto frontier” of the deimmunized design space, comprised of those designs whose sequence and epitope scores are not simultaneously dominated by any other variant (i.e., those designs making the best tradeoffs between the scores). Separate Pepfr runs were performed to identify designs at mutational loads ranging from 1 to 8.

**Figure 2:**
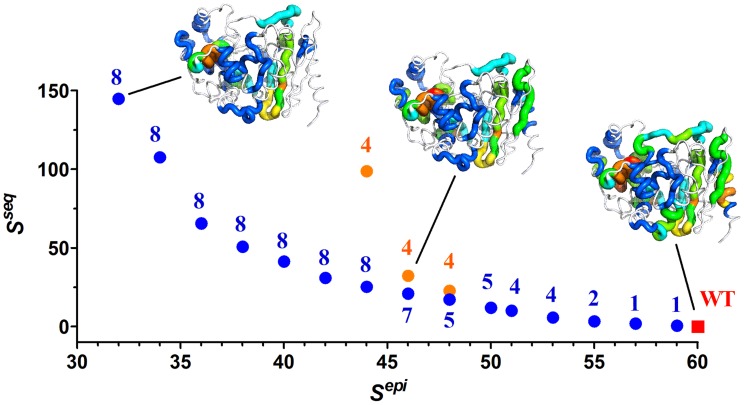
Pareto frontier of the P99βL deimmunized design space. The computed *S^seq^* design parameter is plotted vs. the computed *S^epi^* design parameter for 19 unique enzyme plans. *S^seq^* derives from a statistical sequence potential, and is analogous to an energy function such that lower values are better. *S^epi^* is the total predicted epitope count for each protein. Pareto optimal designs, i.e. those for which no other single design has both better epitope and sequence scores, are indicated with blue circular markers. In orange are three 4-mutation designs that are Pareto optimal at their specific mutational load but are outperformed by designs at higher mutational loads. Wild type P99βL is indicated with a red square. Mutational loads are indicated adjacent to their cognate markers. For three representative proteins, the epitope content has been mapped onto the P99βL peptide backbone (PDB ID 1XX2A). Dense regions of overlapping epitopes are shown as thick red tubes, and lower densities are indicated with incrementally thinner tubes colored in a gradient red-orange-yellow-green-blue. Epitope free regions are thin grey tubes. 3-dimensional epitope maps are shown for wild type P99βL, design 4O, and design 8Z.

The resulting output was a panel of 18 P99βL designs that exhibited a range of mutational loads and extents of epitope disruption. A plot of *S^seq^* vs. *S^epi^* for the 18 protein plans enabled visualization of the objective functions' competing nature (Fig. 2). The overarching goal was reduction of P99βL epitope score via mutagenic deletion of predicted epitopes; however each deimmunizing mutation incurs an *S^seq^* penalty. Any increase above the wild type *S^seq^* reflects a putative risk of reduced protein stability and/or function, and therefore mutagenic deimmunization must carefully balance the opposing objective functions. The Pareto frontier analysis (Fig. 2) highlights the relative tradeoffs between predictions of epitope content and biological activity, but the practical relationship between these mathematical functions is an unknown quantity. Thus, experimental analysis is ultimately required to understand the magnitude of biological activity that is sacrificed per unit immunogenicity.

**Figure 3:**
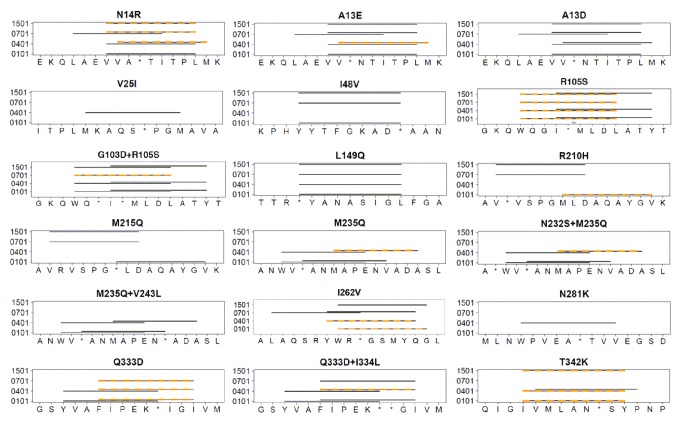
Detailed view of T cell epitopes targeted for disruption. The four MHC II alleles of interest are shown on the y-axis, and peptide sub-sequences of P99βL are shown on the x-axis. Deimmunizing mutations are specified above each graphic, and sites of mutation are indicated by asterisks on the x-axis. The precise positions of predicted T cell epitopes in wild type P99βL are indicated by solid black lines. Predicted epitopes in the specified engineered sequence are indicated with hatched orange lines. Overlapping black and orange lines are predicted epitopes not deleted by the specified mutation or mutations.

Incrementally enhanced deimmunization, moving from right to left on the Pareto curve (Fig. 2), was realized by three complementary mechanisms. First, increasing mutational loads allowed for simultaneous disruption of multiple, distributed epitope clusters. Compare, for example, design 1I, which targets a single epitope with one mutation, to design 8Z, which targets seven distinct immunogenic regions with eight mutations (Table 1). Second, in some instances accrued mutations were combined in close proximity to better target one particularly immunogenic region. For example, designs 4M through 7S as well as plan 8U encoded the R105S mutation, which was predicted to disrupt three of seven epitopes in a dense cluster centered on position 105 (Fig. 3). The more ambitious designs 8V through 8Z deleted six of these same seven epitopes with the combined G103D+R105S double mutation. The mutational combinations M235Q+V243L and Q333D+I334L were likewise predicted to yield enhanced epitope deletion relative to their single mutation counterparts (Fig. 3). In parallel to escalating mutational loads, a third mechanism for improved epitope deletion was the use of increasingly aggressive individual mutations. In particular, mutation N14R was associated with three designs possessing moderate sequence scores (4N, 5R, and 8V; *S^seq^* range of 17.1 to 41.4; Table 1), but it deleted only three of six epitopes in the dense cluster centered on residue 14 (Fig. 3). Mutation A13E, employed by six designs having a *S^seq^* range of 25.3 to 107.6, disrupted five of the six epitopes in this cluster. Finally, A13D deleted all six predicted epitopes, but this aggressive substitution contributed to particularly poor overall sequence scores (*S^seq^* = 98.8 and 144.8 for designs 4P and 8Z, respectively). In aggregate, incremental increases in mutational load and mutational stringency produced a systematic series of deimmunized designs ranging from the wild type *S^epi^* = 60 to that of variant 8Z (*S^epi^* = 32), in which almost half of the predicted epitopes were targeted for disruption.

**Table 1:** Enzyme designs and performance parameters.

Design	A13	N14	K21	V25	I48	G103	R105	R133	L149	R210	M215	N232	M235	V243	I262	N281	Q333	I334	T342	Mut. Load^a^	*S^epi^* Pro-Pred^b^	*S^epi^ IEDB* c	*S^epi^ NN-Align* d	*S^seq^*	k_cat_ e (s^-1^)	K_m_ (µM)	k_cat_/K_m_ (s^-1^ µM^-1^)	T_m_ (°C)	Glob. Imm.^f^ (%)
WT																				0	60	*215*	*791*	0	390±20	44±7	9±1	56.61±0.05	100
1I																			K	1	59	*212*	*795*	0.6	510±90	60±30	9±5	56.27±0.04	82
1J													Q							1	57	*211*	*780*	1.8	420±40	60±10	7±2	55.97±0.03	82
2K										H			Q							2	55	*207*	*779*	3.2	540±70	60±20	9±3	53.14±0.02	62
4L				I						H			Q						K	4	53	*206*	*783*	5.7	370±30	50±10	8±2	53.56±0.03	55
4M							S			H			Q						K	4	51	*198*	*781*	10.0	450±20	51±4	8.7±0.7	50.15±0.02	48
4N		R					S				Q		Q							4	48	*204*	*769*	22.8	500±40	50±10	10±2	49.47±0.04	58
4O	E						S				Q		Q							4	46	*195*	*753*	32.2	460±40	50±10	10±3	49.74±0.04	53
4P	D						S		Q				Q							4	44	*168*	*743*	98.8	250±20	42±7	6±1	50.77±0.02	44
5Q				I			S			H			Q						K	5	50	*200*	*781*	11.9	800±200	80±50	10±6	50.08±0.07	51
5R		R					S			H			Q						K	5	48	*198*	*781*	17.1	640±60	60±20	11±3	49.95±0.04	44
7S				I	V		S			H			Q	L					K	7	46	*201*	*776*	21.0	460±80	90±30	5±2	49.2±0.1	51
8T	E			I	V					H		S	Q			K			K	8	44	*198*	*764*	25.3	430±20	53±8	8±1	54.96±0.05	35
8U	E			I	V		S			H		S	Q			K				8	42	*195*	*758*	30.9	700±80	90±20	8±2	51.54±0.05	35
8V		R		I	V	D	S				Q		Q						K	8	40	*201*	*765*	41.4	310±50	40±20	8±4	50.01±0.05	49
8W	E			I	V	D	S				Q		Q						K	8	38	*191*	*747*	50.8	280±50	40±20	7±4	50.25±0.05	45
8X	E				V	D	S				Q		Q				D	L		8	36	*186*	*746*	65.5	370±10	54±4	6.8±0.6	48.43±0.07	47
8Y	E				V	D	S		Q		Q		Q				D			8	34	*162*	*729*	107.6	300±20	55±7	5.4±0.8	47.21±0.05	39
8Z	D				V	D	S		Q		Q		Q		V					8	32	*163*	*724*	144.8	260±40	50±20	5±2	47.09±0.03	37

a Mutational load - total number of mutations in the specified design.

b Epitope score computed with ProPred at a 5% threshold. Designs were based on these epitope predictions.

c Epitope score computed with IEDB consensus method at a 10% threshold. Predictions were not used for design and are provided for reference only.

d Epitope score computed with NNAlign at a 1000 nM threshold. Predictions were not used for design and are provided for reference only.

e Data are presented as ± SEM from triplicates measured in biological duplicate.

f Global Immunoreactivity is calculated as shown in the materials and methods, equation 4.

### Cloning, Expression, and Purification

Engineered gene constructs were assembled by recursive PCR from overlapping synthetic oligonucleotides, and each gene was modified with a 5′-coding sequence for the OmpA leader peptide and a 3′-coding sequence for a C-terminal hexa-histidine tag. Genes were cloned behind the strong T7 promoter of vector pET26b, and proteins were expressed in the *E. coli* host BL21(DE3) [F^–^ *ompT hsdS_B_* (r_B_^-^ m_B_^-^) *gal dcm* (DE3)]. Recombinant enzymes were released from the periplasm by osmotic shock and subsequently purified to>95% by immobilized metal affinity chromatography. Yields were 1–30 mg/liter of cell culture, depending on the enzyme variant.

### Thermostability Analysis

The relative structural stabilities of the eighteen engineered enzymes were assessed as apparent melting temperatures (T_m_), quantified by differential scanning fluorimetry [34]. The T_m_'s of the eighteen variants ranged from 47.09–56.27°C, or 83–99% of the wild-type value (56.61°C) (Table 1). While the observed 9.5°C range in T_m_ should not be interpreted as insubstantial, it bears noting that none of the engineered variants exhibited significant unfolding at 37°C (S1 Fig.), which is the temperature of ultimate therapeutic relevance.

The incremental manner in which the design series progressively targeted epitopes resulted in extensive mutational overlap between adjacent designs, and insights regarding the destabilizing effects of specific substitutions were obtained by deconvoluting the mutational composition of various constructs. Consider for example the adjacent series 1J, 2K, and 4L. Design 1J encoded only M235Q, which resulted in a negligible 0.64°C reduction in T_m_ (Table 1). In contrast design 2K, which encoded both M235Q and R210H, exhibited a 2.83°C drop in T_m_, indicating that R210H has a significant destabilizing effect, either by itself or in the context of M235Q. The next variant, 4L, revealed that neither V25I nor T342K were destabilizing substitutions, as 4L differed from 2K by only these mutations yet exhibited an equivalent T_m_. The permissible natures of V25I and T342K were further corroborated by comparison of T_m_'s for 5Q vs. 4M and 1I vs. WT, which differed by the respective single mutations and again possessed essentially the same T_m_ values.

Separately, the data indicated that substitutions N14R and A13E were interchangeable with respect to structural integrity. In particular, the 4-mutation designs 4N and 4O differed only by N14R and A13E, respectively, and the 8-mutation variants 8V and 8W exhibited the same distinguishing feature. In both cases, the two alternative substitutions yielded essentially equivalent T_m_ values (49.47≈49.74°C and 50.01≈50.25°C, respectively). Moreover, there was evidence that these N-terminal mutations did not further compromise designs already exhibiting moderately decreased stability. For example, 5R differed from 4M by the simple addition of N14R, yet both enzymes showed similar stability (T_m_ = 50.15 and 49.95°C, respectively).

The most striking observation, however, was the clear bifurcation in T_m_ values between designs that encoded R105S and those that did not. Without exception, plans bearing R105S possessed T_m_'s below 52°C (average T_m_ = 49.53°C), while variants bearing wild type R105 uniformly exhibited T_m_'s above 53°C (average T_m_ = 55.09°C). This suggested that R105S was the single most destabilizing mutation from the study, and separate experiments on the R105S point mutant showed that this single mutation substantially reduced protein stability (T_m_ = 52.64°C, S1 Table). It seems likely that this effect stems from the fact that R105 resides in the center of a pocket defined in part by D86, D87, D108, and E300 (S2 Fig.). Presumably, R105 electrostatically stabilizes the adjacent acidic residues, and removal of this positive charge by the R105S mutation renders the protein less stable. Interestingly, while the isolated R105S mutation caused a reduction in thermostability, it manifested no substantial effect on catalytic activity (S1 Table), and it provided for a net reduction in peptide interaction with human MHC II proteins (see results below). Thus, the unfavorable consequences of R105S appeared to be confined to structural stability.

**Figure 4:**
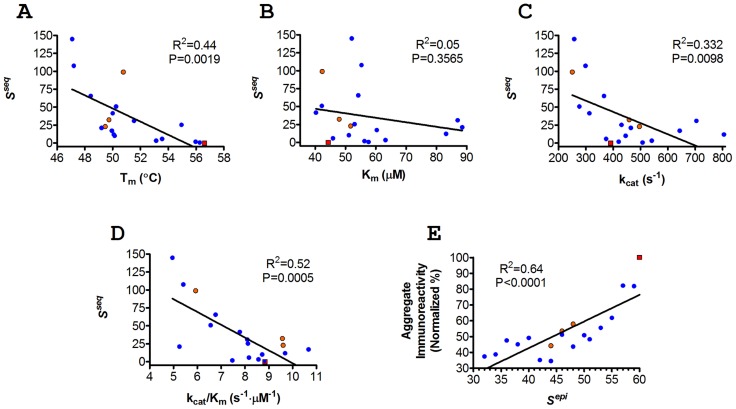
Correlations between computational design parameters and experimentally measured performance metrics. A *S^seq^* vs. T_m_. B *S^seq^* vs. K_m_. C *S^seq^* vs. k_cat_. D *S^seq^* vs. k_cat_/K_m_. E Global Quantitative Immunoreactivity vs. *S^epi^*. (former as defined in equation 4). Pareto optimal enzymes are shown as blue circular markers, sub-optimal 4-mutation variants are shown as orange circular markers, and wild type P99βL is shown as a red square. Linear regressions are shown along with R^2^ values, and an F test was used to determine statistical significance for non-zero slopes (P values are provided).

Finally, it should be noted that the sequence potential was intended, in part, to quantify the likelihood that mutations or combinations of mutations would maintain P99βL structural integrity. A plot of *S^seq^* vs. apparent T_m_ yielded the expected inverse relationship, and a linear regression showed that the correlation was highly significant (non-zero slope, P = 0.0019) (Fig. 4A). While the sequence potential was not an accurate predictor of individual T_m_ values (linear R^2^ = 0.44), from a global perspective it did effectively capture this aspect of experimental performance.

### Kinetic Analysis

A second goal of the sequence potential was to select mutations least likely to disrupt P99βL activity. To assess mutational effects on molecular function, Michaelis-Menten kinetic parameters were quantified using the beta lactam substrate nitrocefin (Table 1). Linear regression of *S^seq^* vs. turnover number (k_cat_) or catalytic efficiency (k_cat_/K_m_) revealed a highly significant inverse correlation (non-zero slope, P = 0.0098 and 0.0005, respectively), whereas there was not a strong correlation with K_m_ (Fig. 4B, C, D). Similar to the relationship with T_m_, the sequence potential could not be used to predict catalytic proficiency for individual enzymes, but it did accurately reflect the overall trends for measured reaction rates and catalytic efficiency.

The k_cat_ values for individual designs ranged from 65–206% that of wild type P99βL (average over all variants = 114%). Notably, 11 of 18 variants exhibited faster than wild type maximum reaction velocities. However, the majority of variants (15/18) also experienced an increase in K_m_, and as a result the average k_cat_/K_m_ for all variants was 88% that of wild type (ranging from 56–121%). In general, wild type or better reaction rates and catalytic efficiencies were maintained up through the two least aggressive 8-mutation plans, 8T and 8U. The two variants with the highest overall k_cat_/K_m_ values were designs 5Q and 5R (109% and 121% of wild type, respectively), and in both cases these enhanced efficiencies were driven exclusively by substantial increases in the k_cat_ parameter (206% and 164% wild type, respectively). Together, these observations highlight the fact that the wild type sequence does not represent a global optimum with respect to catalytic conversion of nitrocefin. The functional deimmunization process identified numerous performance enhanced variants, and the high activity observed across the full spectrum of mutational loads underscores the practical utility of the statistical sequence potential. Indeed, even the five most aggressive 8-mutation designs (8V through 8Z) proved to be highly active enzymes, with k_cat_ and k_cat_/K_m_ values that averaged 77% and 71%, respectively, of wild type. The single most deimmunized variant, 8Z, maintained well above 50% wild type rate acceleration and efficiency. Thus, all 18 deimmunized enzymes exhibited activity comparable to naturally evolved biocatalysts.

### Peptide Binding

**Figure 5:**
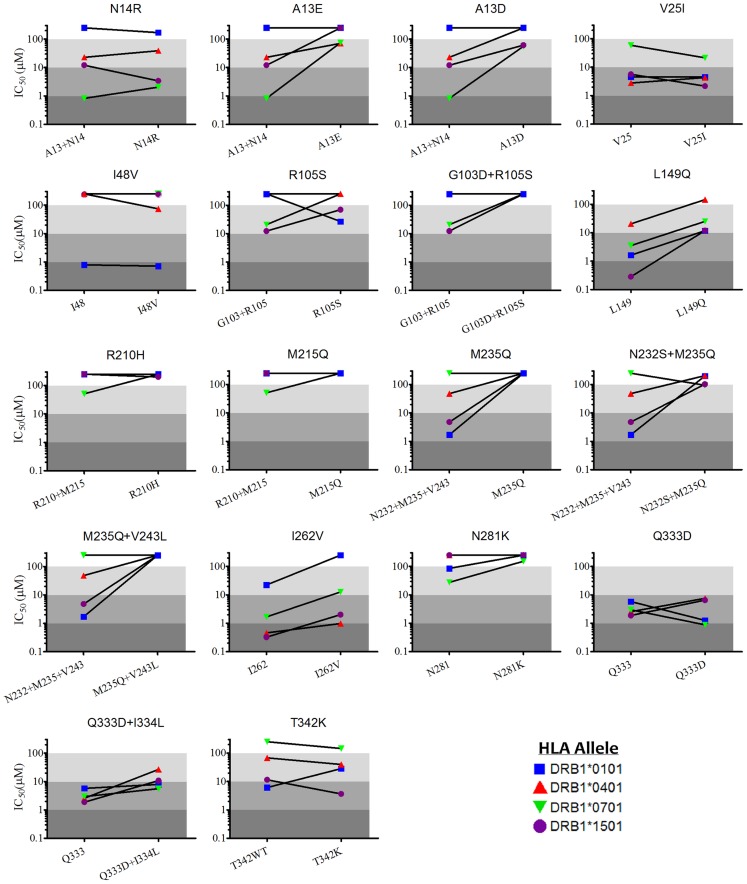
Peptide binding affinities for human MHC II proteins. IC_50_ values are plotted as cognate wild type and variant pairs, where lower IC_50_ values correspond to higher affinity binding with human MHC II. The slope of the connecting lines are a relative measure of deimmunizing efficacy, where larger positive slopes indicate a greater fold decrease in affinity relative to wild type. Lines with negative slopes indicate a mutation that enhanced MHC II binding. Shading indicates binding strength by category: strong (IC_50_<1 µM, dark grey), moderate (1 µM≤IC_50_<10 µM, medium grey), weak (10 µM≤IC_50_<100 µM, light grey), or non-binding (IC_50_ ≥100 µM, white).

The immunoreactivity of various constructs was assessed by measuring the MHC II binding affinity of their corresponding peptide fragments. These competition immunoassays are a widely recognized metric for assessing immunogenic potential and validating computational predictions [22], [25], [33], [35], [36], [37], [38], [39]. Synthetic fragments of wild type P99βL were designed so as to encompass each of the epitopes targeted by the deimmunization algorithm, and corresponding variant peptides were synthesized to represent the deimmunized designs (S2 Table). The affinity of each peptide for human MHC II molecules DRB1*0101, 0401, 0701, and 1501 was measured by competition with known peptide immunogens for each allele. A quantitative comparison of wild type versus variant MHC II binding affinity was used as a proxy measure for the success of epitope deletion (Fig. 5). Peptide affinities are reported as IC_50_ values, and putative epitopes were classified as strong (IC_50_<1 µM), moderate (1 µM≤IC_50_<10 µM), weak (10 µM≤IC_50_<100 µM), or non-binders (IC_50_≥100 µM).

**Figure 6:**
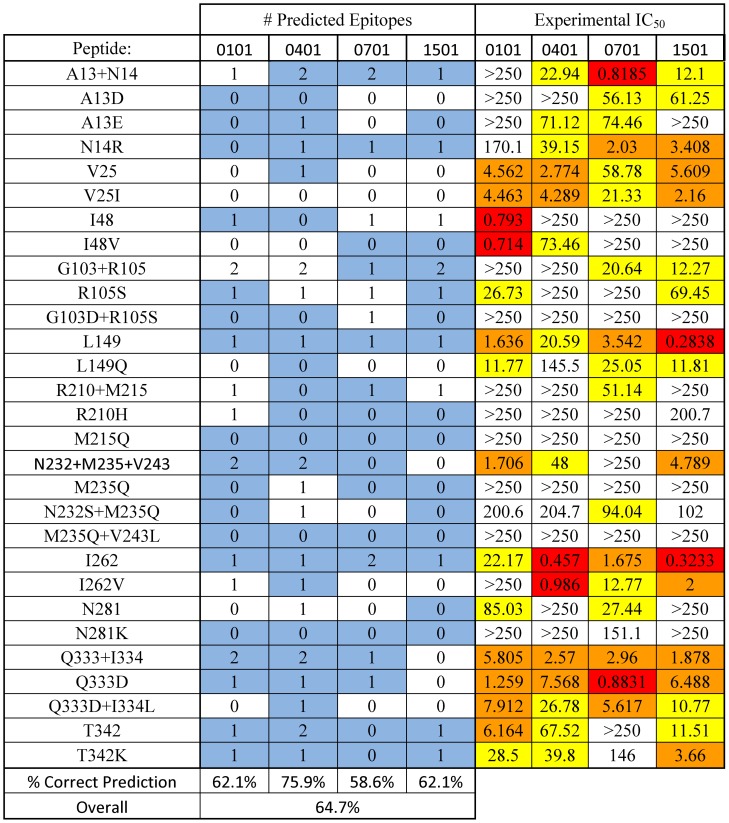
Epitope predictions, measured IC_50_ values, and correlations by individual peptide. For each MHC allele, the number of predicted epitopes within a given synthetic peptide is shown on the left, and the measured IC_50_ values are shown on the right. Peptides were categorized as strong (IC50<1 µM, red), moderate (1 µM≤IC50<10 µM, orange), weak (10 µM≤IC50<100 µM, yellow), or non-binding (IC50 ≥100 µM, white). Positive correlations between epitope prediction and experimental measurements (binding cutoff at 100 µM) are highlighted in blue on the left.

High affinity interaction between peptide antigens and class II MHC is a key determinant of subsequent T cell immunogenicity [40], [41], [42], and a total of four wild type P99βL peptides were found to possess sub-micromolar IC_50_'s for one or more of the tested alleles. The wild type A13+N14 peptide was a high affinity binder of DRB1*0701 (IC_50_ = 800 nM), and both A13D and A13E successfully converted this to a weak binding interaction with N14R yielding a moderate binding interaction (Fig. 6). As found in prior studies [25], wild type peptide L149 was bound by all four alleles, and here it was a particularly strong binder of 1501 (IC_50_ = 300 nM). The L149Q mutation reduced 1501 affinity by 40-fold, converting this strong binding interaction to a weak interaction. Wild type peptide I262 also bound all four alleles, and it possessed sub-micromolar affinity for both 0401 and 1501. The I262V mutation yielded a 6-fold reduction in 1501 affinity, thereby converting a strong binder to a moderate binder. In contrast, I262V did not substantially alter affinity for allele 0401, although this outcome was predicted during the design process (Fig. 3). The only other high affinity binding of a wild type peptide was 0101 binding of I48, which, contrary to predictions, was unaffected by the I48V mutation.

A total of 29 peptides, 11 wild type and 18 engineered, were analyzed to produce 116 affinity measurements. Of the 72 pairs of wild type and cognate deimmunized affinities (Fig. 5), there were 16 cases in which the designed mutation reduced MHC II affinity by more than an order of magnitude. There were an additional 11 instances wherein the designed mutation reduced affinity by 5- to 10-fold, and 10 examples of more modest 2- to 5-fold reductions. In aggregate the engineered mutations showed a 37.5% success rate in reducing MHC II binding by 5-fold or more. In contrast, there were only nine total instances in which the designed mutation enhanced MHC II affinity by any measurable degree. In five of those cases, the increase was a modest 2- to 5-fold, and there were no quantified examples of 10-fold or greater increases in affinity.

To correlate the experimentally measured MHC II affinities with the algorithm's binary prediction of peptide binding/non-binding, a threshold value for experimental “binding” was arbitrarily selected. So as to maintain consistency with our prior work on P99βL, we set the cutoff for experimental binders at an IC_50_<100 µM, i.e. counting all strong, moderate, and weak binders as defined above. Given this experimental threshold and a ProPred prediction threshold of 5%, the protein design process yielded a 65% positive prediction rate for binders across all four alleles (Fig. 6). Predictions were most accurate for DRB1*0401 (76%) and least accurate for allele 0701 (59%). Overall, we observed a 13% false positive rate and a 22% false negative rate, similar to those we have reported previously [25], [31]. Comparable analyses using the newer IEDB consensus [22] and NNAlign [34] prediction methods revealed that, in this instance, no single predictor exhibited dominant accuracy across all four alleles (S3, S4 and S5 Tables). In particular, the ProPred predictor was comparable to the others for the peptides assessed here. As a whole the results show that the IP^2^ deimmunization formulation, implemented through the Pepfr protein design algorithm and using the ProPred epitope predictor, proved to be proficient at identifying high affinity MHC-binding peptides and selecting corresponding disruptive mutations.

**Figure 7:**
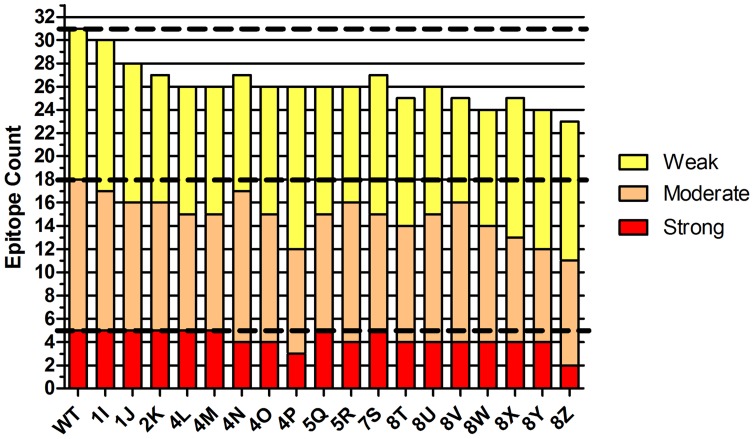
Global categorical immunoreactivity for full length protein designs. The binding strength of individual peptides for MHC II alleles DRB1*0101, 0401, 0701, and 1501 were binned as strong (IC_50_<1 µM, red), moderate (1 µM≤IC_50_<10 µM, orange), weak (10 µM≤IC_50_<100 µM, yellow), or non-binding (IC_50_ ≥100 µM, not shown). The counts for each enzyme's constituent peptides were summed and plotted by semi-quantitative category (y-axis). The horizontal hatched lines are visual guides for the wild type binding counts in each category.

To enable comparison of whole protein immunoreactivity, MHC II binding data for individual peptides was integrated across the full length of each enzyme design. For each protein, a categorical immunoreactivity score was obtained by summing the number of strong, moderate, and weak MHC binders across the protein's component peptides (11 peptides • 4 MHC alleles  =  44 possible interactions). Consistent with the predicted *S^seq^* epitope parameter (Table 1), the design series showed a general trend of decreasing experimental immunoreactivity moving from variant 1I to 8Z (Fig. 7). Of the 18 engineered designs, 11 had a net deletion of one or more high affinity interactions, 17 deleted one or more moderate affinity interactions, and 16 deleted one or more weak interactions. Importantly, none of the engineered enzymes suffered a net increase in total experimental epitopes. In only one case was a design found to have a net addition of epitopes in any single binding category. Namely, 4P possessed one additional weak binder, but at the same time it deleted four moderate and two strong binders, the latter two being most prone to drive a T cell mediated immune response [40]. Indeed, with respect to deleting moderate and strong binders, design 4P was bested only by 8Z. The latter was the single most aggressive design, and it was in fact found to have the lowest categorical immunoreactivity. Specifically, 8Z yielded a net deletion of three strong binders, four moderate binders, and one weak binder, thereby eliminating a full quarter of all experimentally identified MHC II binders. Considering only the higher affinity peptides (IC_50_<10 µM), 8Z benefitted from a 39% reduction in epitope content, similar to the 47% reduction predicted by the deimmunization algorithm. Thus, prediction of epitope disruption was borne out in the overall experimental analysis.

As a second measure of whole-protein immunogenic potential, a global quantitative immunoreactivity value was calculated by averaging the numerical IC_50_'s for each enzyme's component peptides. Importantly, the dynamic range for our MHC II binding assay is 10 nM to 250 µM, and many of the binding affinities, particularly for engineered peptides, were found to be too weak for precise quantitation (values>250 µM, Fig. 6). Because these non-binding peptides are key indicators of reduced immunoreactivity, it was critical that they be factored into the quantitative, whole-protein score. To do so, we employed equation 4 (see materials and methods). Each enzyme's global immunoreactivity, normalized to 100% for wild type P99βL, is reported in Table 1. Similar to the categorical analysis, there was a general trend towards decreased global immunoreactivity with increasing mutational load and aggressiveness. On this normalized scale, designs 8T and 8U are the most immunotolerant variants, both exhibiting a 65% reduction relative to wild type P99βL immunoreactivity. The most extensively engineered design, 8Z, is also highly immunoevasive, with a 63% reduction compared to wild type. Overall, the global, quantitative immunoreactivity was found to have a highly significant and surprisingly close correlation with the predicted *S^epi^* parameter (linear R^2^ = 0.64; non-zero slope P<0.0001; Fig.4E). Thus, *S^epi^* offered reasonable predictive power even for individual P99βL designs. In total, the algorithm successfully incorporated compatible and increasingly effective deimmunizing mutations so as to achieve a systematic reduction in immunogenic potential.

## Discussion

Mutagenic deletion of T cell epitopes, which has been successfully implemented with diverse proteins, is a powerful means for deimmunizing biotherapeutics. With very few exceptions, however, published studies of T cell epitope deletion, in full length proteins, have focused on disrupting one or two immunogenic regions [9], [10], [11], [12], [14], [25], [43]. Indeed, there is debate regarding the feasibility of broad, protein-wide epitope deletion, which is complicated by the high degree of MHC II polymorphism in human populations [44]. Thus, while there are many reports of limited but successful T cell epitope deletion, one is left to wonder how many projects might have failed due to the presence of numerous and dispersed immunogenic regions that could not be targeted simultaneously using conventional strategies. To more fully understand this challenge, we have conducted a combined computational and experimental analysis of the immunogenicity and functionality tradeoffs that are inherent to the deimmunization problem.

**Figure 8:**
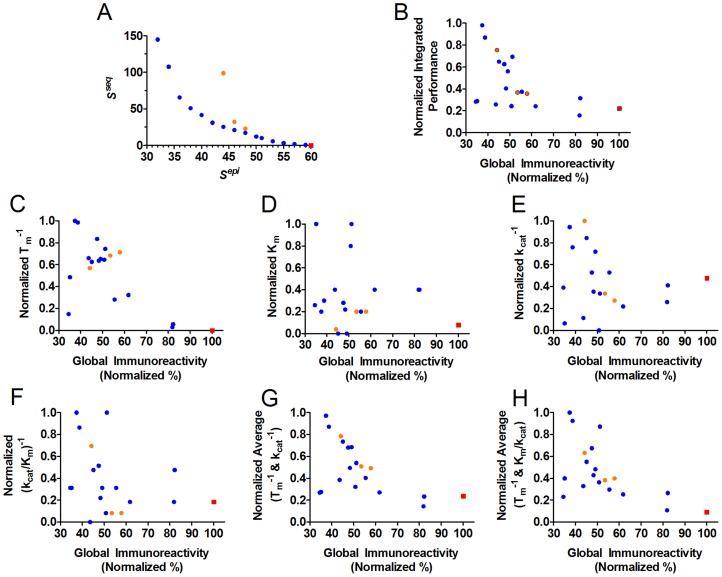
Comparison of computed and experimental Pareto optimal plots. A Pareto plot of computed design parameters *S^seq^* vs. *S^epi^*. B Experimental analog of the computed Pareto plot. An integrated score for experimentally measured molecular function (equation 3) is plotted vs. a global score for experimentally determined immunoreactivity (equation 4). C Experimental Pareto plot of normalized, reciprocal T_m_ vs. Global Quantitative Immunoreactivity. D Experimental Pareto plot of normalized K_m_ vs. Global Quantitative Immunoreactivity. E Experimental Pareto plot of normalized, reciprocal k_cat_ vs. Global Quantitative Immunoreactivity. F Experimental Pareto plot of normalized, reciprocal k_cat_/K_m_ vs. Global Quantitative Immunoreactivity. G Experimental Pareto plot of averaged, normalized, reciprocal k_cat_ and T_m_ vs. Global Quantitative Immunoreactivity. H Experimental Pareto plot of averaged, normalized, reciprocal k_cat_/K_m_ and T_m_ vs. Global Quantitative Immunoreactivity. Pareto optimal enzymes are shown as blue circular markers, sub-optimal 4-mutation variants are shown as orange circular markers, and wild type P99βL is shown as a red square. Note that the computed Pareto plot best captures overall molecular performance, as represented by integrated performance values (e.g. averaging kinetic parameters with thermostability parameters).

The studies described here were enabled by an advanced deimmunization algorithm that seamlessly integrates immunogenic epitope prediction with *in silico* analysis of the functional consequences associated with deimmunizing mutations. We combined the IP^2^ deimmunization formulation with the Pepfr optimization algorithm [28], [32] to design a suite of 18 Pareto optimal P99βL variants. Each of these designs optimally balances two objective functions – one modeling immunogenicity and the other functionality – such that no other single variant is predicted to outperform with respect to both design objectives. Inspection of the Pareto optimal designs reveals that, in the context of the mathematical model, there is an inverse relationship wherein ever greater deimmunization is achieved at the expense of progressively reduced function (Fig. 8A). To assess the practical implications of these predicted tradeoffs, we have recombinantly produced all 18 designed enzymes and rigorously characterized their stability, activity, and immunoreactivity with human MHC II proteins. The results of this analysis represent the first systematic assessment of the functional penalty that is paid for pursuing progressively more deimmunized drug candidates.

Our previous work had demonstrated the capacity to design P99βL variants bearing 1–5 deimmunizing mutations yet retaining wild type or better activity and near wild type stability [25], [31]. By more thoroughly mapping the Pareto optimal design space, we show here that up to seven immunogenic regions can be simultaneously targeted while incurring essentially no loss in molecular stability and function (see design 8T, Table 1). Considering only the most aggressive designs (8T to 8Z), the computational Pareto curve suggested that there would be an accelerating loss of molecular function throughout the series, ultimately resulting in a 6-fold deterioration relative to 8T (Fig. 8A). The experimental analysis verified the predicted trend, revealing that the 8-mutation series did in fact exhibit a systematic reduction in stability and catalytic efficiency. Importantly, however, design 8Z showed a mere 40% reduction in catalytic proficiency relative to 8T. This dramatic difference in the magnitude of Δ*S^seq^* versus measured change in molecular function suggests a non-linear relationship between the statistical sequence potential and actual experimental performance. Indeed, a non-linear correlation is suggested by the above graphical analysis (Fig. 4A, C, D). Thus, it seems likely that more aggressive designs exhibiting even higher *S^seq^* penalties might be realized experimentally before reaching the point of diminishing returns. In other words, it appears we have yet to reach the practical limit of epitope depletion for P99βL.

While the quantified losses in activity and stability were not as sharp as predicted by the *S^seq^* design parameter, there was in fact a general trend towards escalating loss of function with more aggressive deimmunization. To better visualize these real world tradeoffs, we constructed the experimental analog of the Pareto curve (Fig. 8B). This analysis entailed plotting an integrated experimental performance score (averaging the normalized, reciprocal values for T_m_, k_cat_, and k_cat_/K_m_; equation 3) vs. the quantitative global immunoreactivity score (equation 4). The graphical analysis clearly shows that more aggressively deimmunized enzymes sustained progressively greater losses of molecular function. Analogous plots were constructed for various individual performance parameters as well as alternative combinations of these parameters (Fig. 8C-H). It is interesting that the computationally generated Pareto plot most effectively captures the general trends observed with integrated, as opposed to individual, experimental performance measures (compare Fig. 8 panels B, G and H to panels C, D, E and F). This is a notable and advantageous outcome, as biotherapeutic researchers will typically be interested in overall molecular performance as opposed to any single metric (e.g. thermostability, binding affinity, rate acceleration, or catalytic efficiency). As a whole, the parallels between the computational and experimental Pareto plots are striking, and this observation underscores the Pepfr algorithm's capacity to effectively factor in the inherent tradeoffs between immunogenicity and molecular function.

As a final note, previous engineering of P99βL with the IP^2^ algorithm had produced higher activity variants than those designed with the earlier DP^2^ algorithm [31]. However, the IP^2^ designs from the former study were generated after locking down all residues in close proximity the active site. This raised the question of whether or not the combined 1-body + 2-body sequence potential of IP^2^ was in fact more effective than the 1-body potential implemented in DP^2^, where mutations to active site residues had been allowed [25]. The Pareto optimal designs from the present study did not benefit from locked active site residues, yet the 4-mutation and 5-mutation designs from this study substantially outperformed previous 2-mutation DP^2^ designs and were largely equivalent to previous 4-mutation and 5-mutation IP^2^ designs in which the active site had been held invariant (S3 Fig.). This result shows that the more advanced sequence potential of IP^2^ can in fact generate highly mutated and yet highly active proteins in the absence of detailed structure-function information. Moreover, when residues need not be locked down during the design process, there is greater inherent capacity for epitope deletion.

In conclusion, we have computationally and experimentally mapped the deimmunized Pareto frontier of P99βL. The predictions underlying the design process correlated well with experimental analyses of protein function. In particular, we observed that incremental deletion of progressively more T cell epitopes lead to a relative escalation in concomitant loss of function. Thus, the predicted tradeoffs underlying protein deimmunization were borne out in real world analyses. Nonetheless, all 18 of the computationally designed enzymes proved to exhibit reasonable thermostability and impressive activity; not a single design failed to express or function. The most highly engineered enzyme, which incorporated eight mutations targeting seven distinct epitope clusters, was found to have a 39% reduction in high affinity MHC II binding interactions while maintaining well over 50% of the wild type enzyme's catalytic activity. It is therefore evident that we have additional capacity for designing even more extensively deimmunized yet functional P99βL variants. If these trends translate to other therapeutic proteins, as anticipated, the integrated design algorithms evaluated here will accelerate identification of engineered variants spanning a broad spectrum of immunogenic potential and biological function. These panels of deimmunized proteins should prove a rich resource from which to select therapeutic candidates that meet diverse clinical needs.

## materials and methods

### Materials

Oligonucleotides for sequencing and standard PCR methods (25 nmol scale, standard desalting) and oligonucleotides for gene synthesis (100 nmol scale, PAGE Purified) were purchased from Integrated DNA Technology (San Diego, CA). Nitrocefin was purchased from Oxoid (Cambridge, UK). Human lysozyme and SYPRO Orange 5000× Protein Stain were purchased from Sigma (St. Louis, MO). MicroAmp Fast Optical 0.1 ml 96-Well Plates and MicroAmp Optical Adhesive Film were from Applied Biosystems (Bedford, MA). Restriction enzymes and PCR reagents were purchased from New England BioLabs (Ipswich, MA). Growth media was purchased from Becton Dickinson (Franklin Lakes, NJ). Plasmid purification kits and Ni-NTA resin were purchased from Qiagen (Valencia, CA). PCR cleanup and gel extraction kits were from Zymo Research (Irvine, CA). Peptides derived from P99βL were ordered from GenScript (Piscataway, NJ), and were greater than 85% pure. Biotinylated tracer peptides were purchased from 21st Century Biochemicals (Marlborough, MA). MHC II DR molecules were purchased from Benaroya Research Institute (Seattle, WA), anti-MHC II-DR antibody from Biolegend (San Diego, CA), and DELFIA Eu-labeled Streptavidin was from PerkinElmer (Boston, MA). Unless noted, all other chemicals and reagents were from VWR (Radnor, PA).

### Computational Deimmunization

Functionally permissible mutations were identified using an IP^2^ sequence potential, generated essentially as described [28]. Briefly, a multiple sequence alignment (MSA) of 94 homologs from Pfam 00144, including the wild type, was constructed by filtering for ≥30% sequence identity to wild type, ≤90% sequence identity to each other, and ≤25% gaps. The negative log frequency of each amino acid *a* at each position *i* was used to compute position-specific one-body terms φ*_i_*(*a*). Allowed substitutions were constrained to those appearing at or above background amino acid frequencies [45]. Two-body terms φ*_i,j_* (*a*,*b*) for pairs of amino acids (*a*,*b*) at coupled positions (*i*,*j*) were computed as the negative log amino acid frequency of the pair, minus the corresponding one-body terms, which avoids double counting. Only pairs of positions with significant coupling according to a χ^2^-based test were included in the sequence potential. In addition to mutational constraints based on the evolutionary sequence record, prolines and cysteines were neither mutated out of nor substituted into the engineered enzyme variants.

The impact of functionally acceptable mutations on putative T cell epitope content was analyzed with the ProPred epitope predictor set to a 5% threshold. ProPred has been shown to be one of the most accurate MHC II prediction tools in the public space, and readers are referred to the following references for a detailed comparison of different methods [22], [46]. The analysis considered MHC II alleles DRB1*0101, 0401, 0701, and 1501, which are common alleles [42] and for which binding assays had been fully optimized [33]. Each nonamer peptide *X* considered in the optimization (i.e., incorporating a contiguous combination of wild type residues and allowed substitutions) was classified as either a binder or non-binder of the four target MHC II alleles. The number of binders was summed to generate the nonamer's epitope score *e*(*X*). As a comparison, putative epitopes from ProPred predictions were subsequently analyzed using the IEDB consensus [22] and NNAlign [34] prediction methods with binding cutoffs (IEDB 5% or 10%; NNAlign 50 nM or 1000 nM) set to values commonly used in protein immunogenicity prediction [47]. Ultimately, we found that the alternative epitope predictors, when applied to the ProPred based designs, yielded the expected trends of immunogenicity-functionality tradeoffs across the set of 18 P99βL variants (S4 Fig.).

Given a wild-type sequence and a mutational load, Pepfr identifies each Pareto optimal variant *s* with the specified number of mutations making undominated tradeoffs between the competing objectives of total sequence potential *S^seq^* and total epitope score *S^epi^*:



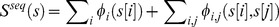
(1)

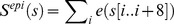
(2)where bracketed expressions indicate selection of the amino acid at the position or the substring of amino acids at the contiguous positions.

Briefly, Pepfr identifies the Pareto frontier of this two-objective space (*S^epi^* vs. *S^seq^*; see Fig. 2) by employing a divide-and-conquer algorithm wrapped around an IP^2^-based variant optimization. Given a region in the objective space (min/max values for the objectives), Pepfr uses a constrained version of IP^2^ to optimize an undominated design in that region. Pepfr then further divides the region into four quadrants around the design and recurses only for the upper-left and lower-right quadrants, as the upper-right is dominated and the lower-left is empty. It thereby finds all and only the Pareto optimal designs, and does so efficiently in that the number of calls to the integer programming optimizer is proportional to the number of Pareto optimal designs. We used Pepfr as described for deimmunization (He et al. 2012), with the underlying integer programming instances optimized by calls to the IBM CPLEX package.

### Cloning, Expression, and Purification

Gene synthesis was performed using a two-step process. First, an assembly reaction was performed using fifty-two synthetic oligonucleotides encoding each design with an appended 5′- *ompA* leader sequence and 3′ hexa-His coding sequence (sequence GGGSAETVEHHHHHH). The assembled genes were then amplified in a second PCR using external primers. The constructs were then digested using *Xba1* and *HindIII*, ligated into similarly digested pET26b, and electroporated into BL21(DE) *E. coli* cells [F^–^ *ompT hsdS_B_* (r_B_^-^ m_B_^-^) *gal dcm* (DE3)].

Expression was performed in 200–500 ml of LB medium containing 30 µg/ml kanamycin (LB-Kan). Expression cultures, from a 1∶100 subculture of saturated overnight cultures, were grown with aeration at 37°C in 2 L baffled flasks for an hour and forty five minutes. The temperature was then shifted to 16°C, equilibrated for 15 minutes, and expression was induced with 1 mM IPTG. Following 12–20 hours of induction at 16°C, osmotic shocktates were prepared using the protocol described in the Epicentre PeriPreps Periplasting Kit with slight modifications. Briefly, cells were pelleted at 6000g for 10 minutes and resuspended in PeriPreps Periplasting Buffer containing 1.5 µg/ml human lysozyme. Cells were quenched after a five minute incubation period with ice-cold water, and then incubated on ice for 10 minutes. The periplasmic fraction was collected by spinning the shocktate at 14,000g for 10 minutes and collecting the supernatant.

Proteins were purified from clarified periplasmic fraction using Ni-NTA resin (400 µl bed volume). After the clarified periplasmic fraction was flowed through the resin by gravity, the column was washed 2 times with 1 mL of PBS (137 mM NaCl, 2.6 mM KCl, 10 mM Na_2_HPO_4_, 1.7 mM KH_2_PO_4_, pH 7.4) containing 20 mM imidazole, and the enzyme was eluted with 2 ml of PBS containing 200 mM imidazole. The elution fraction was either dialyzed (10,000 MW cutoff) against 3 changes of 4 L PBS or concentrated and buffer exchanged by centrifugation (10,000 MW cutoff) against 3 washes of 15 mL PBS to a final concentration of 0.5–2 mg/ml protein. Purified protein was stored at 4°C prior to further analysis. All protein preparations were>95% pure, as determined by reverse-phase HPLC analysis (Agilent 1200 Series HPLC) on a Vydac 214TP 180mm C4 column, eluted at 65°C with a gradient of [90% acetonitrile/9.9% water/0.1% trifluoroacetic acid] in [99.9% water/0.1% trifluoroacetic acid] at a flow rate of 1 ml/min.

### Kinetic Studies

Nitrocefin substrate stock was prepared immediately prior to the experiments by dissolving nitrocefin powder in DMSO to a concentration of 20 mM. Triplicate assays were run in 96-well plate format at 30°C measuring absorbance at 490 nm (Molecular Devices SpectraMax 190 plate reader). Absorbance measurements were converted to micromolar product concentrations using the appropriate molar absorptivity (ε_M_  = 20,500 M^−1^ cm^−1^). The assay buffer was PBS, and each well contained a final enzyme concentration of 50 ng/µl, 0.04% BSA, and nitrocefin at concentrations ranging from 10 µM to 200 µM. Initial reaction rates were plotted against substrate concentration, and Michaelis-Menten kinetic parameters were determined by nonlinear regression using GraphPad Prism v.5 software (La Jolla, CA). Measurements were made in triplicate, and enzymes were purified and assayed in biological duplicate.

### Thermostability

Differential scanning fluorimetry was performed essentially as described (Niesen, Berglund et al. 2007) using an ABI 7500 Fast Real-Time PCR System from Applied Biosystems (Bedford, MA). Proteins and SYPRO Orange were diluted in PBS. Final protein concentrations were 100 µg/ml and final dye concentrations were 5×. Twenty µl reactions were performed in 12 replicates. The PCR gradient was run from 25–94°C with a 1 minute equilibration at each degree centigrade. Fluorescence was quantified using the preset TAMRA parameters. Melting temperatures were determined by data analysis with the “DSF Analysis v3.0.xlsx” Excel sheet (ftp://ftp.sgc.ox.ac.uk/pub/biophysics/) and GraphPad Prism v.5 software.

### Integrated Molecular Performance:

To construct the experimental equivalent of the Pareto optimal plot (Fig. 8), a global molecular performance score was calculated for each individual enzyme using equation 3:
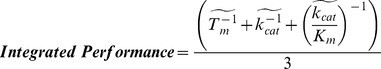
(3)


where “
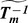
” is the normalized reciprocal T_m_ value, “
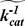
” is the normalized reciprocal k_cat_ value, and “
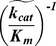
” is the normalized reciprocal k_cat_/K_m_ value. This integrated performance score effectively averages the normalized reciprocal values for thermal stability, rate acceleration, and catalytic efficiency, yielding the experimental analog of S^seq^.

### MHC Binding Assays

MHC II competition binding assays were performed as described [33]. All data were fit to the one-site log(IC_50_) model by non-linear regression in GraphPad Prism v.5 software. Global immunoreactivity values were computed for each variant by (i) averaging the IC_50_ values for all component peptides, (ii) multiplying this figure by the number of peptides with IC_50_>250 µM, (iii) taking the reciprocal of the resulting product, and (iv) taking the ratio of this computed value for a variant to that of wild type P99βL. See equation 4:
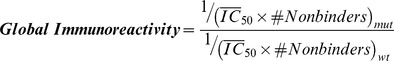
(4)where “
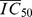
” is the mean IC_50_ value averaged over all component peptides having affinities <250 µM, “#*Nonbinders*” is the total count of component peptides with affinities ≥250 µM, and the subscripts “mut” and “wt” indicate calculations for mutant and wild type proteins, respectively. This calculation accounted for both the affinity of any quantified binders and the equally important metric of total count for non-binders.

### Statistical Analysis

Linear regressions of experimental performance vs. computational predictions employed an F test for statistical significance of non-zero slopes. Significance was determined at the α = 0.05 level.

## Supporting Information

S1 Fig Melting Profiles from differential scanning fluorimetry. Raw fluorescence vs. temperature data from differential scanning fluorimetry is shown. Increased fluorescence correlates with protein unfolding, and T_m_ is computed as the mid-point of the low to high transition. Enzyme designs are indicated above each graph. Note that none of the engineered variants exhibits any measurable unfolding at 37°C.
(TIF)

S2 Fig Local environment of residue R105. The P99βL peptide backbone (PDB ID 1XX2A) is rendered as a grey ribbon, and residues of interest are rendered as van der Waals surfaces. Shown are D86, D87, D108, E300, and in the center R105. Carbon is colored cyan, nitrogen blue, oxygen red, and hydrogen white. The cationic residue R105 sits in a pocket lined by the four acidic residues. Mutation R105S removes the putative stabilizing charge of R105 and may drive protein destabilization through electrostatic repulsion of the four highlighted acidic residues.
(TIF)

S3 Fig Comparison of current and previous P99βL experimental results. The activity and stability of current Pareto optimal P99βL designs has been compared with that of earlier P99βL designs. Values are normalized to the wild type values from the corresponding article. (A) K_m_ value. (B) k_cat_ value. (C) k_cat_/K_m_ value. (D) Apparent T_m_ value. Left of the vertical hashed line are designs from the current study. Pareto optimal enzymes are in dark blue, and sub-optimal enzymes are in yellow. Right of the vertical hashed line in green is a 2-mutation enzyme from an earlier experimentally driven deimmunization program [9], in cyan are 2-mutation enzymes from a previous paper employing the DP^2^ algorithm [25], and in light blue are 4- and 5-mutation enzymes from a previous paper employing the IP^2^ algorithm [31]. Note that 4 and 5-mutation designs from the current study exhibit similar performance to the 4 and 5-mutation designs from the earlier IP^2^ study, despite the fact that the active site residues were locked down in the earlier study but were allowed to mutate here. Additionally, note that the current Pareto optimal designs generally outperform earlier DP^2^ designs, despite the substantially higher mutational loads of most enzymes from the current study.
(TIF)

S4 Fig Predicted sequence scores versus epitope scores using alternative epitope predictors. The IEDB consensus and NNAlign epitope prediction methods were applied to the 18 P99βL designs generated using the ProPred epitope predictor. The expected tradeoffs between epitope score and sequence score manifest the same general trends as in Figure 2. (A) NNAlign based predictions at a 50 nM threshold, (B) IEDB based predictions at a 5% threshold, (C) NNAlign based predictions at a 1000 nM threshold, (D) IEDB based predictions at a 10% threshold. Designs are indicated by name, and wild type is shown as an open red circle.
(TIF)

S1 Table Performance parameters for R105S point mutant.
(DOCX)

S2 Table Synthetic peptides used in MHC II binding studies.
(DOCX)

S3 Table Various epitope prediction methods and correlation with experimental binding.
(PDF)

S4 Table IEDB consensus predictions and correlation with experimental binding.
(PDF)

S5 Table NNAlign predictions and correlation with experimental binding.
(PDF)
