# Supplementary figures and images for: Mapping the Pareto Optimal Design Space for a Functionally Deimmunized Biotherapeutic Candidate
Source: PLoS Comput Biol. 2015 Jan 8;11(1):e1003988. doi: 10.1371/journal.pcbi.1003988 (PMC4288714; doi:10.1371/journal.pcbi.1003988)

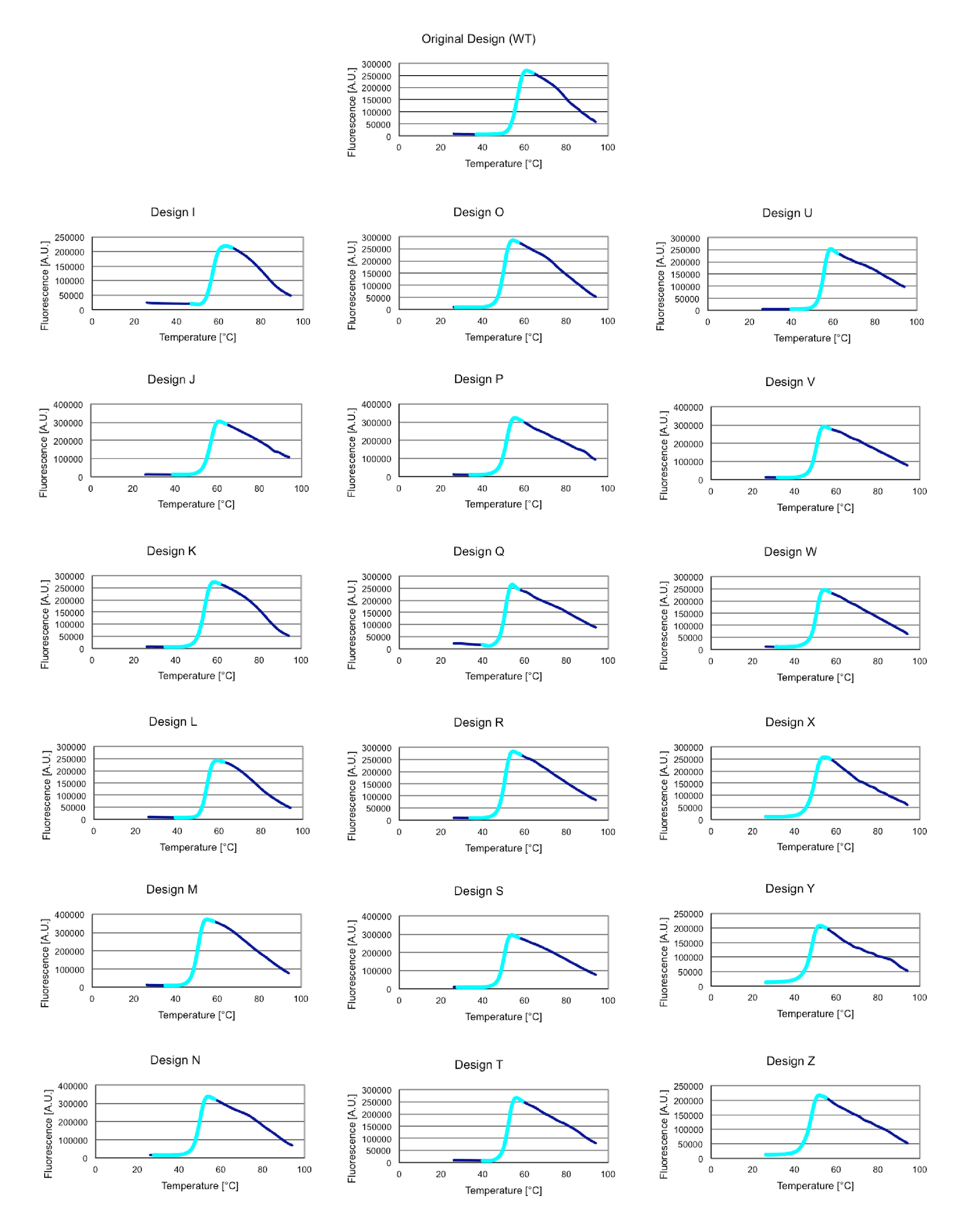

Supplement: S1 Fig — Melting Profiles from differential scanning fluorimetry. Raw fluorescence vs. temperature data from differential scanning fluorimetry is shown. Increased fluorescence correlates with protein unfolding, and Tm is computed as the mid-point of the low to high transition. Enzyme designs are indicated above each graph. Note that none of the engineered variants exhibits any measurable unfolding at 37°C. (TIF) [file pcbi.1003988.s001.tif]

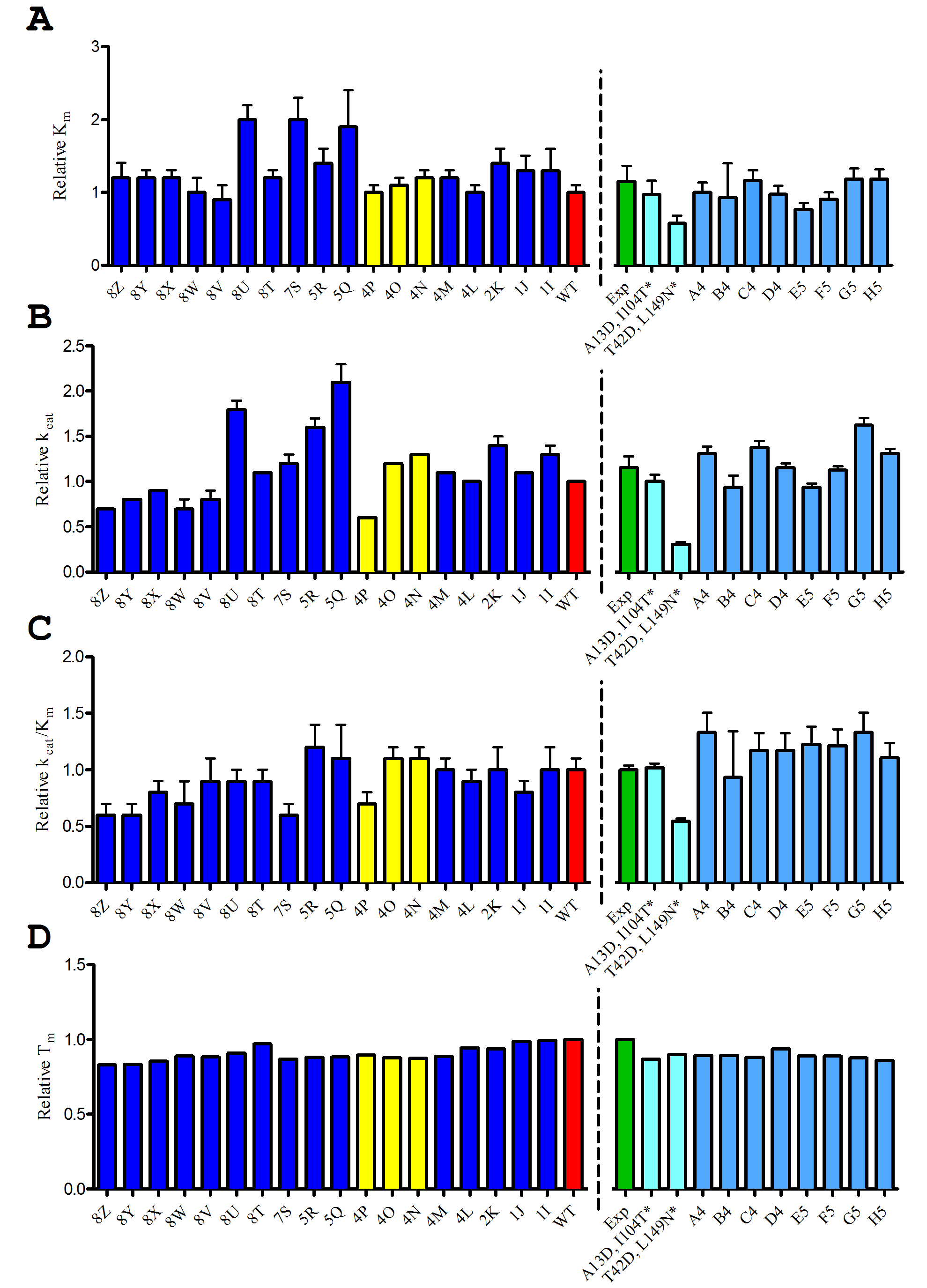

Supplement: S3 Fig — Comparison of current and previous P99βL experimental results. The activity and stability of current Pareto optimal P99βL designs has been compared with that of earlier P99βL designs. Values are normalized to the wild type values from the corresponding article. (A) Km value. (B) kcat value. (C) kcat/Km value. (D) Apparent Tm value. Left of the vertical hashed line are designs from the current study. Pareto optimal enzymes are in dark blue, and sub-optimal enzymes are in yellow. Right of the vertical hashed line in green is a 2-mutation enzyme from an earlier experimentally driven deimmunization program [9], in cyan are 2-mutation enzymes from a previous paper employing the DP2 algorithm [25], and in light blue are 4- and 5-mutation enzymes from a previous paper employing the IP2 algorithm [31]. Note that 4 and 5-mutation designs from the current study exhibit similar performance to the 4 and 5-mutation designs from the earlier IP2 study, despite the fact that the active site residues were locked down in the earlier study but were allowed to mutate here. Additionally, note that the current Pareto optimal designs generally outperform earlier DP2 designs, despite the substantially higher mutational loads of most enzymes from the current study. (TIF) [file pcbi.1003988.s003.tif]

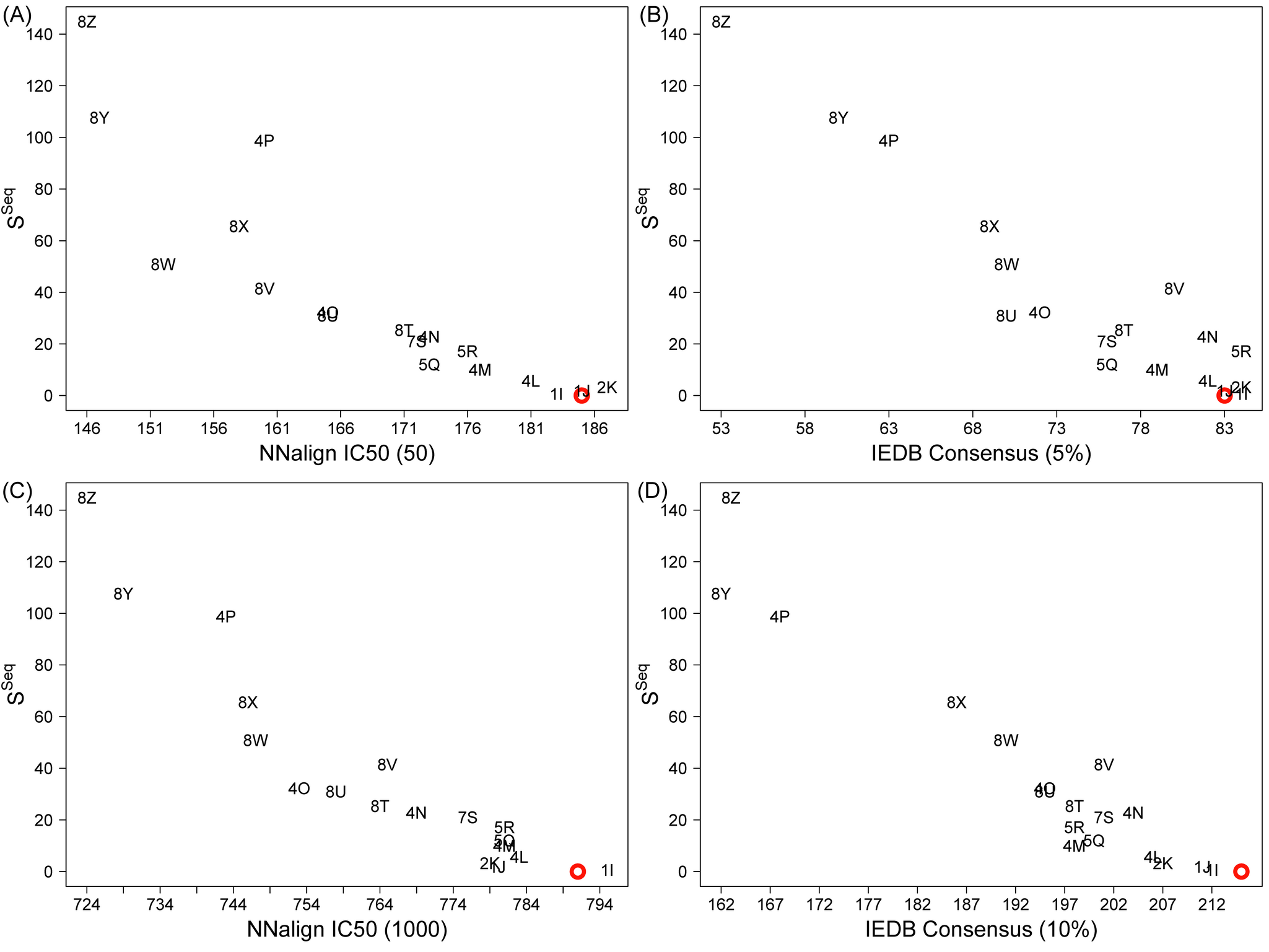

Supplement: S4 Fig — Predicted sequence scores versus epitope scores using alternative epitope predictors. The IEDB consensus and NNAlign epitope prediction methods were applied to the 18 P99βL designs generated using the ProPred epitope predictor. The expected tradeoffs between epitope score and sequence score manifest the same general trends as in Figure 2. (A) NNAlign based predictions at a 50 nM threshold, (B) IEDB based predictions at a 5% threshold, (C) NNAlign based predictions at a 1000 nM threshold, (D) IEDB based predictions at a 10% threshold. Designs are indicated by name, and wild type is shown as an open red circle. (TIF) [file pcbi.1003988.s004.tif]
