# Supplementary material for: Mapping the Pareto Optimal Design Space for a Functionally Deimmunized Biotherapeutic Candidate
Source: PLoS Comput Biol. 2015 Jan 8;11(1):e1003988. doi: 10.1371/journal.pcbi.1003988 (PMC4288714; doi:10.1371/journal.pcbi.1003988)
Supplement: S1 Table — Performance parameters for R105S point mutant. (DOCX) [file pcbi.1003988.s005.docx]

Table S1 – Performance Parameters for R105S Point Mutant

| Design | Mut. Load^a^ | k_cat_^b^ | K_m_ | T_m_ |
| --- | --- | --- | --- | --- |
|  |  | (s^-1^) | (μM) | (ºC) |
| WT R105 | 0 | 390 ± 10 | 82 ± 5 | 56.33 ± 0.03 |
| R105S | 1 | 360 ± 20 | 80 ± 10 | 52.64 ± 0.02 |

a Mutational load - total number of mutations in the specified design

b Data are presented as ± SEM from triplicates measured in biological duplicate
