# Supplementary material for: Mapping the Pareto Optimal Design Space for a Functionally Deimmunized Biotherapeutic Candidate
Source: PLoS Comput Biol. 2015 Jan 8;11(1):e1003988. doi: 10.1371/journal.pcbi.1003988 (PMC4288714; doi:10.1371/journal.pcbi.1003988)
Supplement: S2 Table — Synthetic peptides used in MHC II binding studies. (DOCX) [file pcbi.1003988.s006.docx]

Table S2 – Synthetic peptides used in MHC II binding studies

| **Synthetic Peptide:** | **Amino Acid Sequence** | **Synthetic Peptide:** | **Amino Acid Sequence** |
| --- | --- | --- | --- |
| A13+N14 | EKQLAEVVANTITPLMK | M215Q | AVRVSPGQLDAQAYGVK |
| A13D | EKQLAEVVDNTITPLMK | N232+M235+V243 | ANWVMANMAPENVADASL |
| A13E | EKQLAEVVENTITPLMK | M235Q | ANWVQANMAPENVADASL |
| N14R | EKQLAEVVARTITPLMK | N232S+M235Q | ASWVQANMAPENVADASL |
| V25 | ITPLMKAQSVPGMAVA | M235Q+V243L | ANWVQANMAPENLADASL |
| V25I | ITPLMKAQSIPGMAVA | I262 | GIALAQSRYWRIGSMYQG |
| I48 | KPHYYTFGKADIAAN | I262V | GIALAQSRYWRVGSMYQG |
| I48V | KPHYYTFGKADVAAN | N281 | MLNWPVEANTVVEGSD |
| G103+R105 | GKQWQGIRMLDLATYT | N281K | MLNWPVEAKTVVEGSD |
| G105D+R105S | GKQWQDISMLDLATYT | Q333+I334 | GSYVAFIPEKQIGIVM |
| R105S | GKQWQGISMLDLATYT | Q333D | GSYVAFIPEKDIGIVM |
| L149 | TTRLYANASIGLFGA | Q333D+I334L | GSYVAFIPEKDLGIVM |
| L149Q | TTRQYANASIGLFGA | T342 | QIGIVMLANTSYPNP |
| R210+M215 | AVRVSPGMLDAQAYGVK | T342K | QIGIVMLANKSYPNP |
| R210H | AVHVSPGMLDAQAYGVK |  |  |
