# Supplementary material for: Mapping the Pareto Optimal Design Space for a Functionally Deimmunized Biotherapeutic Candidate
Source: PLoS Comput Biol. 2015 Jan 8;11(1):e1003988. doi: 10.1371/journal.pcbi.1003988 (PMC4288714; doi:10.1371/journal.pcbi.1003988)
Supplement: S3 Table — Various epitope prediction methods and correlation with experimental binding. (PDF) [file pcbi.1003988.s007.pdf]

**Table S3. Various Epitope Prediction Methods and Correlation with Experimental Binding**[a]				
**Prediction Method and Predictive Binding Threshold**	**Fraction Correct**[b]			
	DRB1*0101	DRB1*0401	DRB1*0701	DRB1*1501
**Propred 5% Threshold**	0.62	0.76	0.59	0.62
**IEDB Concensus 5% Threshold**	0.52	0.76	0.59	0.52
**IEDB Concensus 10% Threshold**	0.55	0.83	0.59	0.66
**NNAlign 50 nM Threshold**	0.76	0.62	0.66	0.48
**NNAlign 1000 nM Threshold**	0.52	0.76	0.69	0.90

a Predicted binding at the specified threshold was compared to experimental binding at a 100 μM threshold

b Fraction correct predictions are taken from Figure 6, Table S4, and Table S5
