# Supplementary material for: Mapping the Pareto Optimal Design Space for a Functionally Deimmunized Biotherapeutic Candidate
Source: PLoS Comput Biol. 2015 Jan 8;11(1):e1003988. doi: 10.1371/journal.pcbi.1003988 (PMC4288714; doi:10.1371/journal.pcbi.1003988)
Supplement: S4 Table — IEDB consensus predictions and correlation with experimental binding. (PDF) [file pcbi.1003988.s008.pdf]

Table S4. IEDB Concensus Predictions and Correlation with Experimental Binding												
	DRB1*0101			DRB1*0401			DRB1*0701			DRB1*1501		
Peptide	percentile[a]	correct5?[b]	correct10?[c]	percentile	correct5?	correct10?	percentile	correct5?	correct10?	percentile	correct5?	correct10?
A13+N14	13.08	1	1	5.06	0	1	3.43	1	1	12.99	0	0
A13D	50.05	1	1	10.5	1	1	16.92	0	1	43.37	0	0
A13E	45.58	1	1	5.12	0	1	12.86	0	1	33.64	1	1
N14R	15.98	1	1	4.42	1	1	4.16	1	1	12.35	0	0
V25	10.18	0	0	1.86	1	1	3.59	1	1	14.56	0	0
V25I	10.53	0	0	3.11	1	1	5	0	1	9.11	0	1
I48	25.85	0	0	8.55	1	0	29.17	1	0	32.59	1	1
I48V	20.67	0	0	6.95	0	1	39.66	1	0	27.1	1	1
G103+R105	23.77	1	1	5.09	1	0	7.48	0	1	9.3	0	1
R105S	26.51	0	0	8.55	1	0	14.67	1	0	14.35	0	0
G103D+R105S	34.74	1	1	13.12	1	1	23.74	1	0	18.19	1	1
L149	2.91	1	1	6.72	0	1	3.75	1	1	9.84	0	1
L149Q	21.6	0	0	19.73	1	1	12.46	0	1	26.59	0	0
R210+M215	21.75	1	1	27.26	1	1	26.33	0	1	29.15	1	1
R210H	22.89	1	1	27.41	1	1	24.44	1	0	29.12	1	1
M215Q	46.1	1	1	49.99	1	1	25.57	1	0	48.62	1	1
N232+M235+V243	27.97	0	0	9.4	0	1	36.71	1	0	27.83	0	0
M235Q	50.05	1	1	16.25	1	1	38.25	1	0	44.32	1	1
N232S+M235Q	43.54	1	1	15.92	1	1	38.25	0	1	48.71	1	1
M235Q+V243L	50.05	1	1	16.13	1	1	38.25	1	0	45.55	1	1
I262	20.67	0	0	13.24	0	0	2.93	1	1	4.42	1	1
I262V	24.27	1	1	18.43	0	0	5.02	0	1	7.3	0	1
N281	42.36	0	0	35.63	1	1	60.4	0	1	39.85	1	1
N281K	59.93	1	1	47.75	1	1	62.51	1	0	49.86	1	1
Q333+I334	23.11	0	0	2.36	1	1	11.52	0	1	14.45	0	0
Q333D	28.09	0	0	4.36	1	1	17.95	0	1	17.92	0	0
Q333D+I334L	25.47	0	0	3.93	1	1	15.88	0	1	17.21	0	0
T342	7.73	0	1	0.42	1	1	27.19	1	0	4.09	1	1
T342K	11.83	0	0	2.44	1	1	34.26	1	0	2.16	1	1
Total Correct		15	16		22	24		17	17		15	19
Fraction Correct		0.52	0.55		0.76	0.83		0.59	0.59		0.52	0.66

a Percentile score from IEDB concensus prediction method

b Correlation of binding prediction with experiment using a prediction threshold of ≤5% and experimental threshold of 100 µM. Correct=1; Incorrect=0

c Correlation of binding prediction with experiment using a prediction threshold of ≤10% and experimental threshold of 100 µM. Correct=1; Incorrect=0
