# Supplementary material for: Mapping the Pareto Optimal Design Space for a Functionally Deimmunized Biotherapeutic Candidate
Source: PLoS Comput Biol. 2015 Jan 8;11(1):e1003988. doi: 10.1371/journal.pcbi.1003988 (PMC4288714; doi:10.1371/journal.pcbi.1003988)
Supplement: S5 Table — NNAlign predictions and correlation with experimental binding. (PDF) [file pcbi.1003988.s009.pdf]

Table S5. NNAlign Predictions and Correlation with Experimental Binding												
	**DRB1*0101**			**DRB1*0401**			**DRB1*0701**			**DRB1*1501**		
Peptide	IC50[a]	correct50?[b]	correct1000?[c]	IC50	correct50?	correct1000?	IC50	correct50?	correct1000?	IC50	correct50?	correct1000?
A13+N14	23.9	0	0	195.8	0	1	18.1	1	1	579.3	0	1
A13D	511.1	1	0	614.4	1	0	132.6	0	1	4956.3	0	0
A13E	326.5	1	0	724.7	0	1	86.4	0	1	2640	1	1
N14R	31.5	0	0	94.9	0	1	21.5	1	1	420.1	0	1
V25	8.1	1	1	29.6	1	1	18.9	1	1	583.2	0	1
V25I	9.1	1	1	42.7	1	1	25.9	1	1	448.1	0	1
I48	15.3	1	1	105.6	1	0	373.5	1	0	1577.4	1	1
I48V	19.5	1	1	85.6	0	1	754.6	1	0	1661.3	1	1
G103+R105	27.1	0	0	63.9	1	0	41.8	1	1	182.9	0	1
R105S	30	1	1	139	1	0	105.4	1	0	567.7	0	1
G103D+R105S	108.2	1	0	171.2	1	0	244.6	1	0	773.4	1	0
L149	7.3	1	1	91.1	0	1	59.3	0	1	110.2	0	1
L149Q	51.5	0	1	434.5	1	0	358.6	0	1	392	0	1
R210+M215	52.1	1	0	2422.4	1	1	302	0	1	1379.7	1	1
R210H	57.2	1	0	3055.3	1	1	259.3	1	0	1986.4	1	1
M215Q	267.7	1	0	7802	1	1	1052.9	1	1	3900	1	1
N232+M235+V243	126.3	0	1	1159	0	0	1520.1	1	1	1911.8	0	0
M235Q	598	1	0	1735.7	1	1	1407.9	1	1	6260.3	1	1
N232S+M235Q	478.2	1	0	1470.8	1	1	1821.5	0	0	7702.3	1	1
M235Q+V243L	453.2	1	0	1580.6	1	1	1257.8	1	1	5809.3	1	1
I262	26.9	1	1	383.1	0	1	15.6	1	1	25.2	1	1
I262V	37.9	0	0	498.9	0	1	26	1	1	30.6	1	1
N281	151	0	1	3622.5	1	1	2459.3	0	0	3743.5	1	1
N281K	238.6	1	0	6695.8	1	1	2751.3	1	1	2338.1	1	1
Q333+I334	24.9	1	1	101.3	0	1	73.4	0	1	417.1	0	1
Q333D	37.5	1	1	117.3	0	1	146.7	0	1	696.6	0	1
Q333D+I334L	19.9	1	1	105.2	0	1	119.5	0	1	643.9	0	1
T342	13.6	1	1	12.2	1	1	322.8	1	0	82	0	1
T342K	21.1	1	1	35.5	1	1	534.1	1	0	59.1	0	1
**Total Correct**		22	15		18	22		19	20		14	26
**Fraction Correct**		0.76	0.52		0.62	0.76		0.66	0.69		0.48	0.90

a IC_{50} value from NNAlign prediction method

b Correlation of binding prediction with experiment using a prediction threshold of $IC_{50} \leq 50$ nM and experimental threshold of 100 μM. Correct=1; Incorrect=0

c Correlation of binding prediction with experiment using a prediction threshold of $IC_{50} \leq 1000$ nM and experimental threshold of 100 μM. Correct=1; Incorrect=0
